# Harnessing 3D Scanning and Printing Technology to Improve Students’ Proficiency in Assessing Foot Posture

**DOI:** 10.1002/jfa2.70056

**Published:** 2025-06-20

**Authors:** Daniel R. Bonanno, Sheree E. Hurn, Helen A. Banwell, Daniel Alizzi, Hylton B. Menz

**Affiliations:** ^1^ Discipline of Podiatry School of Allied Health Human Services and Sport La Trobe University Victoria Australia; ^2^ School of Clinical Sciences Queensland University of Technology Kelvin Grove Australia; ^3^ Allied Health and Human Performance University of South Australia Adelaide Australia

**Keywords:** education, foot posture, podiatry, simulated learning, students, university

## Abstract

**Introduction:**

The Foot Posture Index (FPI‐6), widely used to quantify foot posture, is a core component of musculoskeletal curricula in undergraduate podiatry programs. Teaching the FPI‐6 can be challenging but 3D foot models provide a controlled risk‐free way to practice, potentially reducing anxiety and increasing confidence. This study examined the effects of 3D foot models on podiatry students' confidence and anxiety when performing the FPI‐6 and compared their scores to experts.

**Methods:**

Fifty podiatry students from three Australian universities used the FPI‐6 to score nine 3D printed foot models ranging from −11 (highly supinated) to +12 (highly pronated). Students' self‐confidence and anxiety were measured before and after exposure to the 3D foot models using a 10‐item self‐confidence questionnaire and the 27‐item Competitive State Anxiety Inventory‐2 (CSAI‐2). Changes in self‐confidence were analysed with paired *t*‐tests, whereas median differences in CSAI‐2 scores pre‐ and post‐intervention were assessed using the Wilcoxon signed‐rank test. Students' foot posture scores were compared to consensus scores from an expert panel (*n* = 4) with variability in agreement explored using the Bland–Altman limits of agreement (LoA) analysis.

**Results:**

Student confidence improved across all 10 questionnaire items after the FPI‐6 simulation with 3D foot models (*p* ≤ 0.015) with a mean increase of 8.6% across all items (range, 1.9%–11.6%) and medium to large effect sizes (Cohen's *d* = 0.44–0.94). On the CSAI‐2, 22 of 27 items showed improvements in cognitive and somatic state anxiety or self‐confidence (*p* ≤ 0.038), whereas five items showed no significant change. The Bland–Altman analysis revealed a small mean difference of 0.389 between student and expert consensus scores with 95% LoA ranging from −3.3 to 4.1.

**Conclusion:**

The use of 3D foot models for FPI‐6 simulation enhances podiatry students' confidence and reduces anxiety. Student's foot posture scores had good overall agreement with expert scores, though some discrepancies remained. This highlights the value of pre‐scored models for targeted practice and emphasises the importance of validation and feedback to ensure confidence aligns with accuracy. The models demonstrated high utility, harnessing 3D scanning and printing technology to enhance students' proficiency in assessing foot posture.

**Abbreviations**
FPI‐6: Foot Posture Index ‐ 6
LTU: La Trobe University
QUT: Queensland University of Technology
STL: Stereolithography
UNISA: University of South Australia

## 1 Introduction

The Foot Posture Index (FPI‐6) is an observational clinical assessment tool for quantifying standing foot posture [1]. It is commonly used by podiatrists and other healthcare professionals to assess and manage a wide variety of foot and lower limb conditions [2, 3]. As a result, the FPI‐6 is a core component of biomechanics and musculoskeletal assessment curricula in many podiatry programs, both in Australia and globally.

The FPI‐6 uses six validated observations to score a person's foot posture [1]. Each of the six observations are scored on a 5‐point scale (−2 to +2) which are summated to produce a score that can range from −12 (highly supinated) to +12 (highly pronated) [1]. The most common FPI‐6 score is reported to be +4 (slightly pronated) with 68% of the population having a score within +1 to +7 [4]. Accordingly, podiatry students are less likely to observe less common foot postures outside this range during their studies.

The FPI‐6 has demonstrated good to excellent intra‐examiner reliability across various user experience levels [5, 6] and excellent intra‐ and inter‐examiner reliability when translated into multiple languages [7, 8]. Although these studies have supported the reliability of the FPI‐6, they have primarily evaluated foot postures within a range that reflects more commonly observed foot types [5, 6, 7, 8]. Notably, podiatry students were not included in previous reliability studies, and it is uncertain how well their assessments agree with those of experienced assessors across a wider range of FPI scores.

Assessing foot posture may be challenging and potentially anxiety‐provoking for podiatry students as it requires integrating palpatory skills with complex anatomical, morphological and mechanical concepts. Anxiety can be highly detrimental to the learning process [9], particularly for students during the early stages of skill development [10] or when attempting rare or challenging tasks [9]. Confidence plays a crucial role in managing anxiety with higher levels of confidence helping to reduce the negative effects of anxiety on performance [11, 12]. Managing anxiety and building confidence in students can be achieved by offering learning opportunities in environments that foster success without the risk of failure [12]. Clinical simulations replicate real‐world scenarios in controlled environments, and are commonly used in medical and allied health education [13, 14] to facilitate acquisition of clinical skills through repeated practice without negative consequences [12].

Two studies evaluated the use of 3D simulation in podiatry settings, focusing on scalpel debridement, demonstrating improvements in self‐confidence and competence and reduced anxiety among students [15, 16]. These positive findings highlight the potential of 3D foot models for simulated teaching in other areas such as performing FPI‐6 as part of musculoskeletal assessments [17]. Doing so could further validate the broader utility of 3D simulations in podiatry education and support their integration into diverse clinical teaching contexts. As with the scapel debridement studies [15, 16], 3D simulations can potentially assist students in improving confidence and reducing anxiety while also providing opportunities to verify competence [17]. The latter can be achieved by assessing students' performance against established outcomes or expert benchmarks [17].

The primary aim of this study was to evaluate the effects of simulated learning of the FPI‐6 using 3D printed foot models, created from original scans of diverse foot postures, on student confidence and anxiety. The secondary aim was to compare FPI‐6 scores obtained by students to consensus scores by experienced podiatrists.

## 2 Materials and Methods

### 2.1 Study Design

A quasi‐experimental pre‐post study design was conducted with podiatry students from three Australian universities (La Trobe University [LTU], University of South Australia [UniSA] and Queensland University of Technology [QUT]). The study examined changes in students' confidence and anxiety before and after practicing the FPI‐6 using 3D printed foot models. Students' FPI‐6 scores were also compared to an expert consensus to determine agreement between podiatry students and experienced podiatrists. Ethical approval was provided by the LTU Human Ethics Committee (HEC23048) with institutional approval also obtained from the UniSA (206211) and QUT (8290). All participants provided informed consent prior to data collection.

### 2.2 Participants

Undergraduate podiatry students were recruited in August 2024. Students from relevant year‐level cohorts were invited to participate through poster advertisements sent via email. To be eligible to participate, students needed to have been first taught the FPI‐6 within the previous 12 months as part of their undergraduate programme, be at least 18 years old and be proficient in English. Participation was voluntary, and all participants provided written informed consent before enrolment. Potential participants were assured that non‐participation or withdrawal at any time would not result in any negative consequences.

### 2.3 Outcome Measures

#### 2.3.1 Self‐Confidence

Self‐confidence in using the FPI‐6 was measured with a modified version of a questionnaire previously used to assess podiatry students' confidence regarding their scalpel debridement [15] which demonstrated excellent internal reliability (Cronbach’s alpha = 0.94). The questionnaire consisted of 10 questions with questionnaire wording modified to focus on the FPI‐6 as the skill of interest. Six questions addressed the six criteria of the FPI‐6, whereas the remaining questions focused on overall performance using the FPI‐6, scoring the FPI‐6 and positioning of both the student and the patient. The questionnaire used a visual analogue scale (VAS) to score each of the 10 questions where 0% represented ‘no confidence at all’ and 100% represents ‘as confident as you have ever felt’.

#### 2.3.2 Anxiety

Competitive State Anxiety Inventory‐2 (CSAI‐2) was used to measure anxiety [18, 19]. CSAI‐2 consists of 27 questions across three subscales: somatic state anxiety (physiological symptoms of anxiety such as increased heart rate and clammy hands), cognitive state anxiety (worry and negative thoughts) and self‐confidence [18, 19]. CSAI‐2 uses an ordinal 4‐point Likert subscale where 1 represents ‘not at all’ and 4 represents ‘very much so’ [19]. CSAI‐2 is a validated tool for measuring self‐reported anxiety, commonly used in sports performance settings [18, 20] and has demonstrated internal consistency in a previous study involving undergraduate podiatry students (self‐confidence domain Cronbach’s alpha = 0.75) [15]. Participants were instructed to report their feelings in the present moment, imagining they were about to perform an FPI‐6 assessment on a client.

### 2.4 Procedure

#### 2.4.1 Foot Scans and 3D Printed Foot Models

Foot scans were sourced from an existing library from previous scanning studies conducted by the Discipline of Podiatry at LTU (ethics number: HEC18269). All participants had provided written informed consent for their scans to be used in future projects. 3D scans of feet and distal lower legs (height of 20 cm from the ground) were obtained with participants in a relaxed bipedal standing position using the INFOOT High Type Laser Scanner (I‐Ware Laboratory Co. Ltd, Japan). The scanner is accurate within 1.0 mm with measurements validated against plain film X‐rays and demonstrated to be reliable in healthy individuals [21]. Foot scans were stored as stereolithography (STL) files.

Each foot was scored using the FPI‐6 prior to scanning. DRB and HBM initially screened and selected foot scans representing a range of foot postures, from highly supinated to highly pronated, while also ensuring good representation of the five scores (−2 to +2) across the six FPI‐6 scoring criteria. In addition, foot scans were selected based on several factors including high scan quality (minimal blemishes from the scanning process), absence of significant foot deformities (apart from high‐arched or low‐arched feet), participants aged 18 years or older, no history of foot surgery and a balanced representation of sex, age and left and right feet.

Fifteen STL files were initially selected to represent a diverse range of FPI‐6 scores; participant sex and foot laterality with scan quality also considered. Although 25 foot posture scores exist (ranging from −12 to +12), using a file for each was deemed overly burdensome for students and offered limited additional educational value as differences between adjacent scores are often subtle. The initial selection of 15 files was made with the intention of potentially refining the set further, balancing educational value with practical workload considerations. These files were then sent to an additive manufacturing device (i.e., 3D printer) to create solid foot models made from polylactic acid (PLA), a low‐cost widely available biodegradable, and bioactive thermoplastic derived from renewable organic resources. Three sets of 3D printed foot models were created with one set provided to each of the three participating universities.

#### 2.4.2 Consensus FPI‐6 Scoring of Foot Models

**FIGURE 1:**
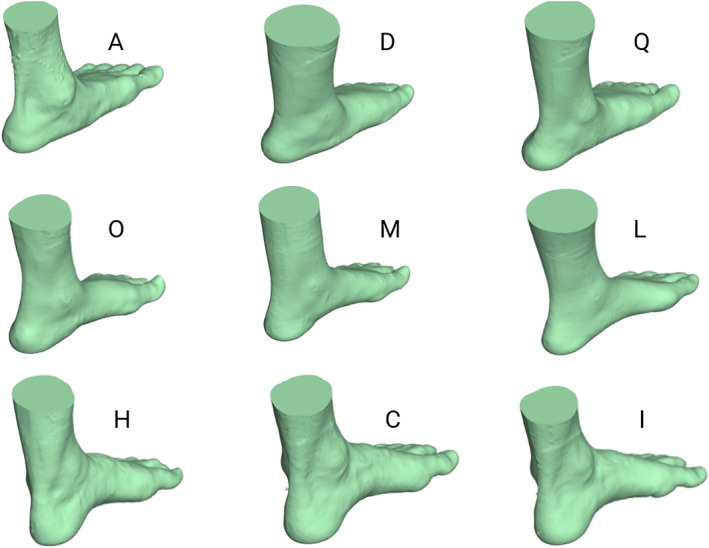
SLT files of the selected foot models. NB: right foot models mirrored to the left foot to improve visualisation.

An expert panel of four academic podiatry staff members (DRB, SEH, HAB and HBM) from the three participating universities met via Zoom to score the 3D foot models using the FPI‐6 [22]. All panel members were registered podiatrists with a minimum of 15 years of experience in publishing and teaching within the field of biomechanics and musculoskeletal conditions affecting the foot and ankle. The total number of models was reduced from 15 to 9 to make it easier to complete and minimise fatigue among participants (see Supporting Information S1: Appendix 1 for STL files of the nine 3D foot scans). These nine 3D foot models provided a range of scores for each of the six FPI‐6 criteria. Scores ranged from −2 to +2 for all criteria, except for the supra‐ and infra‐malleolar curvature, where a score of −2 was not represented because of the lack of a suitable scan exhibiting this characteristic. The nine selected foot models represented FPI‐6 scores from −11 to +12 and included five male and four female models with five left and four right feet (Figure 1).

#### 2.4.3 Student Simulated Learning Session

The student simulated learning sessions used the same methods at all three universities. Relevant university–course–level coordinators emailed students from relevant year levels to inform them about the study and provided details about data collection dates, times and locations. Data collection sessions were held in classrooms at the three respective university campuses with participants attending a single session each. Multiple timeslots were offered on days the students attended in‐person classes for the convenience of participation.

Each session began with an overview of the project. If students were eligible and provided consent to participate, they then completed a paper‐based pre‐test questionnaire on self‐confidence and anxiety related to performing the FPI‐6 (see ‘Outcome measures’). A standardised instructional video (Supporting Information S1: Appendix 2) was then played to the attending participants on how to perform and score the FPI‐6 as described by Redmond and colleagues [1, 22]. Students then individually assessed each of the nine 3D foot models using the FPI‐6 while being able to refer to the user guide as needed [22]. The models were randomly assigned letters (e.g., A, C, D or H) unrelated to their FPI scores. Each 3D foot model was placed on a separate desk in alphabetical order, spaced about 1 m apart. Participants started at any available desk and were given 3 minutes per foot model to perform the FPI‐6. Every 3 minutes, they rotated to the next desk, continuing this process until all models were assessed. Students were requested to perform under exam‐like conditions (i.e., no collaborating or talking). After finishing performing the FPI‐6 on each of the nine 3D foot models, students completed their post‐test questionnaire on self‐confidence and anxiety. In total, each session lasted approximately 1 hour.

### 2.5 Statistical Analysis

Data analysis was performed using IBM SPSS 29.0 statistical software (IBM Corp, Armonk NY, USA), except for the Bland–Altman analysis which was conducted using Stata 18.0 (StataCorp LLC, College Station, TX, USA). Data were explored for normality, and none required transformation. Paired *t*‐tests were used to compare mean values of the self‐confidence questionnaire before and after the intervention. Cohen's *d* was calculated to determine the magnitude of this difference [23] with effect sizes interpreted as follows: very small ≤ 0.01, small ≥ 0.2, medium ≥ 0.5, large ≥ 0.8, very large ≥ 1.2 and huge ≥ 2.0 [24]. A Wilcoxon signed‐ranked test was performed for the ordinal data of the CSAI‐2 to investigate the median differences pre‐ and post‐intervention [25]. Variability in agreement between students and experts was examined using the Bland–Altman analysis [26]. Statistical significance was set at *p* < 0.05 for all tests [25].

## 3 Results

### 3.1 Participant Characteristics

A total of 51 students participated across the three universities: 15 from UniSA, 15 from LTU and 21 from QUT. However, one participant from UniSA was excluded from the study as a statistical outlier because of suspected errors or misunderstanding in completing the forms, resulting in 50 participants being included in the data analysis. Most participants were female (68%), the mean age was 24.9 years (ranging from 20 to 51) and there was a broad ethnicity profile (Table 1).

**TABLE 1:** Participant characteristics (*n* = 50).

Female	34 (68)
Age in years [mean (SD), range]	24.9 (6.2), 20 to 51
Institution of study, year level	
La Trobe University, year 3	15 (30)
University of Queensland, year 3	21 (42)
University of South Australia, year 3	14 (28)
Ethnicity	
Oceanian	17 (34)
North‐West European	3 (6)
South and Eastern European	4 (8)
North African and Middle Eastern	1 (2)
South‐East Asian	7 (14)
North‐East Asian	8 (16)
Southern and Central Asian	7 (14)
Peoples of the Americas	1 (2)
Sub‐Saharan African	2 (4)
Australian Aboriginal or Torres Strait Islander persons	0 (0)

*Note:* Values are frequency (%) unless stated.

### 3.2 Effects on Self‐Confidence

A summary of the self‐confidence questionnaire results is provided in Table 2. Student confidence improved significantly across all 10 questions following the FPI‐6 simulation with the 3D foot models (*p* ≤ 0.015). The post‐test mean improvement in confidence across all questions was 8.6%, ranging between 1.9% and 11.6%, with effect sizes categorised as medium to large (*d* = 0.44–0.94). The largest improvement was observed for the overarching question (item 1) ‘*How confident are you in your ability to perform FPI‐6?*’ with a mean difference of 11.6% and a large effect size (*d* = 0.94). In contrast, the smallest improvement was recorded for the question (item 7), ‘*How confident are you in your ability to appropriately score bulging of the talo‐navicular joint in the region of the sustentaculum tali?*’ with a mean difference of 1.9% and a medium effect size (*d* = 0.44).

**TABLE 2:** Confidence performing FPI‐6 (0%–100%)^a^ pre‐ and post‐exposure to 3D foot models (*n* = 50).

Item	Pre‐test	Post‐test	Effect size^b^	*p*‐value^c^
1. How confident are you in your ability to perform FPI‐6?	66.9 (13.5)	78.7 (11.5)	0.94 (large)	< 0.001^*^
2. How confident are you in your ability to appropriately position the patient?	73.8 (16.8)	83.0 (14.6)	0.58 (medium)	< 0.001^*^
3. How confident are you in your ability to appropriately position yourself?	75.9 (14.8)	86.7 (11.4)	0.82 (large)	< 0.001^*^
4. How confident are you in your ability to appropriately score talar head palpation?	71.3 (14.9)	77.6 (13.9)	0.44 (small)	0.015^*^
5. How confident are you in your ability to appropriately score supra‐ and infra‐ lateral malleoli curvature?	63.8 (14.1)	74.0 (16.5)	0.66 (medium)	< 0.001^*^
6. How confident are you in your ability to appropriately score calcaneal frontal plane position?	76.7 (15.1)	86.5 (10.3)	0.76 (medium)	< 0.001^*^
7. How confident are you in your ability to appropriately score bulging of the talo‐navicular joint in the region of the sustentaculum tali?	71.5 (15.9)	78.3 (14.9)	0.44 (small)	0.005^*^
8. How confident are you in your ability to appropriately score congruence of the medial longitudinal arch?	76.4 (17.2)	86.6 (10.8)	0.71 (medium)	< 0.001^*^
9. How confident are you in your ability to appropriately score abduction/adduction of the forefoot on the rearfoot?	81.9 (14.7)	90.1 (9.7)	0.66 (medium)	< 0.001^*^
10. How confident are you in your ability to appropriately score overall foot posture when using the FPI‐6?	72.7 (13.5)	81.6 (10.9)	0.73 (medium)	< 0.001^*^

*Note:* Values are mean (SD) unless stated.

^a^ Zero percent represents ‘no confidence at all’ and 100% represents ‘as confident as you've ever felt’.

^b^ Cohen's *d*.

^c^ Paired sample *t*‐test.

^*^ significant difference at *p* < 0.05.

### 3.3 Effects on Anxiety

**FIGURE 2:**
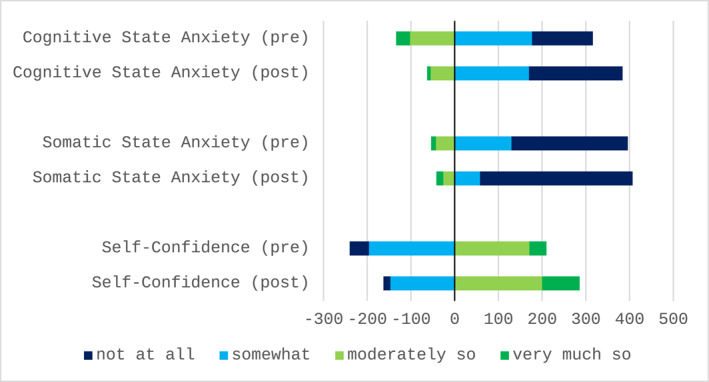
Total responses to the Competitive State Anxiety Inventory‐2 for cognitive state anxiety, somatic state anxiety and self‐confidence: pre‐ and post‐simulated learning (*n* = 50). Cognitive state anxiety score is based on summating results from items 1, 4, 7, 10, 13, 16, 19, 22 and 25; somatic state anxiety score is based on summating results from items 2, 5, 8, 11, 14, 17, 20, 23 and 26; self‐confidence score is based on summating results from items 3, 6, 9, 12, 15, 18, 21, 24 and 27; negative scores (to the left of 0) indicate a negative result and positive scores (to the right of 0) indicate a positive result.

The CSAI‐2 responses for cognitive state anxiety, somatic state anxiety and self‐confidence subscales, measured before and after simulated learning, are summarised in Figure 2 with a detailed breakdown of student scores for each item available in Appendix 3. Of the 27 questions on the CSAI‐2, students demonstrated statistically significant reductions in cognitive state anxiety, somatic state anxiety or self‐confidence for 22 questions following FPI‐6 simulation with 3D foot models. The remaining five questions showed no significant change between pre‐ and post‐test results.

All nine questions related to cognitive state anxiety demonstrated statistically significant improvements (*p* ≤ 0.038) with a reduction in the summated median pre‐to post‐intervention scores from 17 to 14 (lower scores indicating better outcomes). Six out of nine questions related to somatic state anxiety demonstrated statistically significant improvements with a reduction in the summated median pre‐to post‐intervention scores from 12 to 11 (lower scores indicating better outcomes). Seven of the nine questions related to self‐confidence demonstrated statistically significant improvements with an increase in the summated median pre‐to post‐test scores from 22 to 27 (higher scores indicating better outcomes).

### 3.4 FPI‐6 Scores of Students and Experts

A summary of student FPI‐6 scores compared to expert consensus are presented in Table 3. The Bland–Altman analysis revealed a small mean difference (bias) of 0.389 between the two groups, indicating a high level of overall agreement. The 95% LoA were −3.3 to 4.1, indicating that most differences lie within this range and are likely around the mean difference, suggesting good agreement between the students' and expert consensus scores (see Appendix 4 for the Bland–Altman plot comparing student and expert scores).

**TABLE 3:** FPI‐6 scores in students (*n* = 50) and experts (*n* = 4).

Foot model	Student mean (SD), range	Expert consensus
A	9.9 (1.7), 5 to 12	12
C	−4.1 (2.7), −9 to 2	−6
D	6.9 (2.1), 1 to 11	8
H	−5.6 (3.1), −11 to 10	−5
I	−7.2 (2.4), −11 to 0	−11
L	1.1 (1.2), −1 to 4	1
M	3.5 (2.1), −3 to 8	3
O	4.8 (2.1), −1 to 10	6
Q	9.2 (2.1), 3 to 12	7

*Note:* The Bland–Altman analysis revealed that the mean difference (bias) for the student scores versus the expert consensus scores was 0.389, and the limits of agreement ranged from −3.3 to 4.1.

## 4 Discussion

This study examined the effects of 3D printed foot models on podiatry students' confidence and anxiety when performing the FPI‐6 while also comparing their scores to those of experienced podiatrists. The use of 3D foot models in simulated learning not only increased students' confidence and reduced their anxiety but also resulted in a good level of agreement between student and expert FPI‐6 scores, highlighting the effectiveness of this teaching method.

Following the use of 3D foot models, participants reported medium to large increases in confidence (Cohen's *d* ≥ 0.5) on all 10 questions of the self‐confidence survey. Similarly, seven of the nine questions related to self‐confidence on the CSAI‐2 demonstrated statistically significant improvements with the summated median pre‐to post‐intervention score increasing from 22 to 27. Notably, these gains occurred despite students already reporting relatively high confidence in performing the Foot Posture Index‐6 (FPI‐6) prior to the simulation.

However, changes in anxiety, as measured by the CSAI‐2, were less pronounced. The relatively low pre‐test anxiety levels, particularly somatic anxiety, limited the ability to detect substantial changes following simulated learning. This may be partly explained by the CSAI‐2 being designed for high pressure contexts, such as sports [18, 19], whereas our study did not induce such high anxiety levels. In our study, summated median scores improved by 9 points on a 108‐point scale following the 3D simulation. Subscale changes included a 5‐point increase in self‐confidence, a 3‐point decrease in cognitive state anxiety and a 1‐point decrease in somatic state anxiety.

Since the minimum detectable change for the CSAI‐2 is not established, its sensitivity remains uncertain. However, the standard error of measurement ranges from 1.6 to 2.6 across subscales in preadolescent female gymnasts, suggesting changes of 1.6 or less may reflect measurement error [27]. Although our population differs, applying this range to our data suggests changes exceeding 2.6 are more likely to represent true differences. Therefore, the 5‐point improvement in self‐confidence is likely meaningful, the 3‐point decrease in cognitive state anxiety may indicate a small benefit, whereas the 1‐point change in somatic state anxiety is unlikely to be meaningful.

Promoting confidence and lowering anxiety are essential for a safe learning environment but fostering clinical competence is equally important. In our study, podiatry students with less than 12 months of FPI‐6 experience showed good agreement with experienced podiatrists' scores. In a practical context, the Bland–Altman mean difference (bias) of 0.389 indicates that, on average, students' scores are very close to the experts' scores. The 95% LoA ranging from −3.3 to 4.1 show that most of the differences between student and expert scores fall within this range. This indicates that while students generally score similarly to experts, there is some variability, and the range in foot postures scores indicates low agreement for some students. Using pre‐scored 3D foot models helps identify these students for additional practice, providing a low‐risk opportunity to refine their clinical skills. This is crucial if students feel confident but are not accurate, as it could lead to over‐confidence and errors in clinical decision‐making. This underscores the need for ongoing validation exercises and targeted feedback in simulation tasks to ensure that increased confidence is paired with improved accuracy.

The good agreement of FPI‐6 scores in our study between podiatry students and experienced podiatrists is comparable to findings from other studies involving allied health students assessing foot posture on real people such as osteopathy students (ICC = 0.85–0.86) [5] and physiotherapy students (ICC = 0.92–0.94) [28]. Overall, our findings support the use of 3D foot models for teaching students to score foot posture, with students' results showing high agreement with experienced podiatrists, comparable to scores obtained on real feet in previous studies.

Our findings align with theories explaining *why* learners gain confidence and *how* the learning environment supports this process [12, 29]. By providing realistic and constructive experiences in a safe and controlled setting, without fear of consequence, our study observed an improvement in task‐specific confidence [12, 29]. Such approaches reduce inherent anxieties and foster self‐efficacy, preparing students for clinical scenarios involving unfamiliar tasks such as scoring rare foot postures [12, 29]. Our findings build on previous research demonstrating that simulated learning, both broadly and when specifically using 3D foot models (e.g., scalpel use for foot ulcers), is beneficial in podiatry education settings by enhancing student confidence, self‐efficacy and patient safety [15, 16, 17]. Considering this, there is a strong rationale for expanding simulation‐based training into higher‐stakes areas, such as wound management and nail surgery, where students may experience heightened anxiety and reduced confidence.

### 4.1 Strengths and Limitations

This study has two main strengths. Firstly, this study recruited podiatry students from three universities from different states in Australia. This not only accounted for variations in curricula but also ensured a diverse and representative sample with broad demographics. This diversity enhances the generalisability of our findings while minimising location‐ and university‐specific biases. Consequently, the observed improvements in self‐confidence and reductions in anxiety are likely applicable to undergraduate podiatry programs at other universities. Secondly, we used scans obtained from a diverse range of participants to create 3D foot models, and the corresponding STL files can be used to 3D print these models for any university teaching the FPI‐6. This enhances the scalability of our findings and ensures their global applicability in podiatry education.

This study has two main limitations. Firstly, this study used 3D foot models made from PLA, a firm thermoplastic polyester, which likely affects the accurate completion of any FPI‐6 criteria requiring soft‐tissue palpation because of the absence of soft tissue simulation. Specifically, the inability to differentiate between foot veins, muscles (e.g., abductor hallucis) and bone via palpation made assessing talo‐navicular congruence and talar head prominence challenging. Consequently, self‐confidence in these two FPI‐6 components showed the smallest improvement, indicating students' relative difficulty in simulating these tasks compared to those requiring only visual observation. Secondly, the quasi‐experimental design of the study lacked a control group, limiting our ability to definitively determine whether the observed improvements in anxiety and self‐confidence were solely because of exposure to the 3D foot models or if they simply resulted from additional learning of the FPI‐6 through the instructional video. The absence of a control group also limits our ability to determine whether this method is superior to standard practice which typically involves peer assessment in a teaching setting (e.g., tutorials, skills laboratories etc.). As such, we cannot rule out the possibility that other factors, such as additional practice or the natural progression of the students' skills, may have contributed to the improvements. The medium to large effect sizes in anxiety and self‐confidence, along with students' ability to assess rare foot postures, suggest that exposure to the 3D foot models likely enhanced proficiency in the FPI‐6. However, future studies with a control group are needed to better isolate the models' effects.

## 5 Conclusion

The use of 3D printed foot models for simulated learning of the FPI‐6 significantly improves self‐confidence and reduces anxiety levels in podiatry students. These foot models offer a novel approach to teaching, enabling students to encounter rare extremes of the FPI‐6 spectrum that are not typically observed during their undergraduate studies. Additionally, podiatry students demonstrated excellent agreement with experienced podiatrists in scoring the FPI‐6 on the 3D foot models. Overall, the findings support the utility of incorporating 3D foot models into university education for teaching foot posture assessments.

## Author Contributions

**Daniel R. Bonanno:** conceptualization, funding acquisition, investigation, methodology, data curation, formal analysis, supervision, project administration, visualization, writing – original draft. **Sheree E. Hurn:** conceptualization, funding acquisition, investigation, methodology, data curation, project administration, visualization, writing – review and editing. **Helen A. Banwell:** conceptualization, funding acquisition, investigation, methodology, data curation, project administration, visualization, writing – review and editing. **Daniel Alizzi:** methodology, formal analysis, visualization, writing – original draft. **Hylton B. Menz:** conceptualization, funding acquisition, investigation, methodology, data curation, formal analysis, supervision, project administration, visualization, writing – original draft.

## Ethics Statement

Ethics approval was obtained by the La Trobe University Human Ethics Committee (reference number HEC23048) with mutual acceptance also obtained from the University of South Australia (206211) and Queensland University of Technology (8290). Written informed consent was obtained from all participants before taking part.

## Consent

The authors have nothing to report.

## Conflicts of Interest

HBM and SEH serve as Trustees of APERF. Additionally, SEH is a Director of ACPD. These roles have been fully disclosed and appropriately managed to uphold the integrity of this research.

## Supporting information

Supporting Information S1

Supporting Information S2

Supporting Information S3

Supporting Information S4

Supporting Information S5

Supporting Information S6

Supporting Information S7

Supporting Information S8

Supporting Information S9

Supporting Information S10

Figure S1

Table S1

Movie S1

## Data Availability

All STL files of foot scans used in this study are available in Supporting Information S1: Appendix 1. All other data are available upon reasonable request of the authors.
