# Supplementary material for: Harnessing 3D Scanning and Printing Technology to Improve Students’ Proficiency in Assessing Foot Posture
Source: J Foot Ankle Res. 2025 Jun 20;18(2):e70056. doi: 10.1002/jfa2.70056 (PMC12179434; doi:10.1002/jfa2.70056)
Supplement: Supplementary file 1 — Supporting Information S1 [file JFA2-18-e70056-s011.docx]

Appendix 1 : SLT files of foot models for 3D printing

Appendix 2 : FPI-6 instructional video: performing and scoring guide
