# Supplementary figures and images for: Harnessing 3D Scanning and Printing Technology to Improve Students’ Proficiency in Assessing Foot Posture
Source: J Foot Ankle Res. 2025 Jun 20;18(2):e70056. doi: 10.1002/jfa2.70056 (PMC12179434; doi:10.1002/jfa2.70056)

Appendix 4. Bland-Altman plot comparing student and expert scores


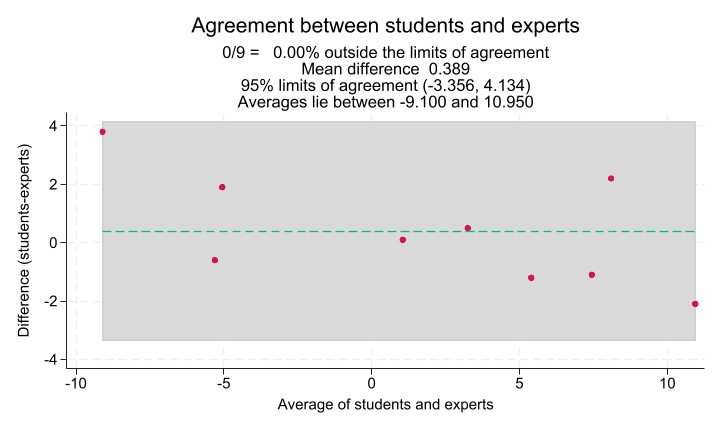

Supplement: Supplementary file 11 — Figure S1 [file JFA2-18-e70056-s005.docx]
