# Supplementary material for: Harnessing 3D Scanning and Printing Technology to Improve Students’ Proficiency in Assessing Foot Posture
Source: J Foot Ankle Res. 2025 Jun 20;18(2):e70056. doi: 10.1002/jfa2.70056 (PMC12179434; doi:10.1002/jfa2.70056)
Supplement: Supplementary file 12 — Table S1 [file JFA2-18-e70056-s004.docx]

Appendix 3. Competitive State Anxiety Inventory 2 scores (*n* = 50)

| **Item** | **Pre-test** | **Post-test** | ***p*-value** |
| --- | --- | --- | --- |
| 1. I am concerned about using FPI-6^a^ | 2.00 (1.00) | 1.00 (1.00) | 0.003* |
| 2. I feel nervous^a^ | 2.00 (1.00) | 1.00 (1.00) | < 0.001* |
| 3. I feel at ease^b^ | 2.00 (1.00) | 3.00 (2.00) | 0.001* |
| 4. I have self-doubts^a^ | 2.00 (1.00) | 2.00 (0.25) | 0.003* |
| 5. I feel jittery^a^ | 1.00 (1.00) | 1.00 (0.00) | < 0.001* |
| 6. I feel comfortable^b^ | 3.00 (1.00) | 3.00 (2.00) | 0.010* |
| 7. I am concerned I may not do as well using as I should^a^ | 3.00 (1.00) | 2.00 (2.00) | < 0.001* |
| 8. My body feels tense^a^ | 1.00 (1.00) | 1.00 (0.00) | 0.032* |
| 9. I feel self-confident^b^ | 2.00 (1.00) | 3.00 (1.00) | 0.008* |
| 10. I am concerned about not performing as well as my colleagues^a^ | 2.00 (2.00) | 2.00 (1.25) | 0.001* |
| 11. I feel tense in my stomach^a^ | 1.00 (0.25) | 1.00 (0.00) | 0.020* |
| 12. I feel secure^b^ | 2.00 (1.00) | 3.00 (1.00) | 0.065 |
| 13. I am concerned about choking under pressure^a^ | 1.00 (1.00) | 1.00 (0.00) | 0.002* |
| 14. My body feels relaxed^b^ | 3.00 (1.00) | 3.00 (2.00) | 0.093 |
| 15. I'm confident I can meet the challenge^b^ | 3.00 (1.00) | 3.00 (0.25) | 0.079 |
| 16. I'm concerned about performing poorly^a^ | 2.00 (1.00) | 2.00 (1.00) | 0.002* |
| 17. My heart is racing^a^ | 1.00 (1.00) | 1.00 (0.00) | 0.002* |
| 18. I'm confident about performing well^b^ | 2.00 (1.00) | 3.00 (1.00) | 0.004* |
| 19. I'm worried about reaching adequate proficiency with performing FPI-6^a^ | 2.00 (0.25) | 2.00 (1.00) | 0.038* |
| 20. I feel my stomach sinking^a^ | 1.00 (0.00) | 1.00 (0.00) | 0.058 |
| 21. I feel mentally relaxed^b^ | 3.00 (1.00) | 3.00 (1.25) | 0.013* |
| 22. I'm concerned that others will be disappointed with my performance^a^ | 2.00 (1.00) | 1.00 (1.00) | 0.028* |
| 23. My hands are clammy^a^ | 1.00 (1.00) | 1.00 (0.00) | 0.022* |
| 24. I'm confident because I mentally picture myself reaching my goal^b^ | 2.00 (1.00) | 3.00 (1.00) | 0.001* |
| 25. I'm concerned I won't be able to concentrate^a^ | 1.00 (1.00) | 1.00 (0.25) | 0.016* |
| 26. My body feels tight^a^ | 1.00 (0.00) | 1.00 (0.00) | 0.071 |
| 27. I'm confident of coming through under pressure^b^ | 3.00 (1.00) | 3.00 (1.00) | 0.002* |

Cognitive State Anxiety items are: 1, 4, 7, 10, 13, 16, 19, 22 and 25; Somatic State Anxiety items are 2, 5, 8, 11, 14, 17, 20, 23, and 26; Self-Confidence items are: 3, 6, 9, 12, 15, 18, 21, 24 and 27

Values are median (interquartile range) unless stated

^a^high score represents a negative result

^b^high score represents a positive result

^*^significant difference at *p* < 0.05
